# Epidermoid Cyst of the Soft Palate in a Geriatric Cat: A Case Report

**DOI:** 10.3390/vetsci13101064

**Published:** 2026-10-09

**Authors:** Hyehyun Choi, Kyung Ah Kim, Da Eun Lee, Juwon Lee, Jihun Kim, Hyomi Jang, Dong-In Jung

**Affiliations:** 1 24h Jamsil ON Animal Medical Center, Seoul 05556, Republic of Korea; veteong@gmail.com (H.C.); vetdaeun@naver.com (D.E.L.); juwon008@naver.com (J.L.); vetmgm@hanmail.net (J.K.); 2 Seocho I:UM Animal Hospital, Seoul 06643, Republic of Korea; vetkatekim@gmail.com; 3 Academic Committee, Korean Society of Feline Medicine (KSFM), Seoul 08531, Republic of Korea; 4 College of Veterinary Medicine, Gyeongsang National University, Jinju 52828, Republic of Korea

**Keywords:** epidermoid cyst, soft palate, computed tomography, surgical excision, cat

## Abstract

Epidermoid cysts within the oral cavity are rare in cats. Here, we report the clinical, imaging, surgical, and histopathological findings of an epidermoid cyst originating from the soft palate of a geriatric cat. A 17-year-old neutered female domestic shorthaired cat presented with mild hypersalivation and dysphagia. Computed tomography revealed a well-circumscribed, minimally contrast-enhancing mass measuring 8.5 × 9.9 × 10.0 mm arising from the right distal soft palate. The mass protruded predominantly into the oropharynx and caused mild nasopharyngeal displacement, without evidence of bone invasion or middle ear involvement. Surgical excision was performed, and histopathological examination revealed a cyst lined with keratinizing stratified squamous epithelium containing keratinaceous debris, which was consistent with a benign epidermoid cyst. Hypersalivation and dysphagia were no longer observed after excision, although this improvement was documented for only approximately 24 h before severe gastrointestinal dysfunction and recurrent seizures developed. Although repeated oral mucosal irritation was considered a possible contributing factor, the congenital or acquired origin of the cyst could not be definitively determined. This case highlights that epidermoid cysts should be considered in the differential diagnosis of cystic lesions of the oral cavity in cats.

## Simple Summary

Epidermoid cysts are uncommon benign cystic lesions in cats, and their occurrence in the soft palate is rarely reported. In this case, a soft palate mass was identified by computed tomography in a 17-year-old cat and confirmed histopathologically as an epidermoid cyst after surgical excision. Hypersalivation and dysphagia were no longer observed during the short postoperative period, although this temporal association does not establish causation. The cat subsequently developed severe gastrointestinal dysfunction, electrolyte disturbances, and recurrent seizures and died 27 days after surgery. The origin of the cyst remains uncertain, with both developmental and acquired mechanisms possible. This case highlights the clinical and diagnostic features of a soft palate epidermoid cyst in a geriatric cat.

## 1. Introduction

Oral masses account for approximately 3–12% of all masses in cats, and >89% are malignant [1,2,3]. In contrast, oral cysts are uncommon and histologically characterized by fluid- or semi-fluid-filled cavities lined by epithelium [4,5]. Among the various types of oral cysts, odontogenic cysts are relatively well-documented in cats. These cysts arise from remnants of odontogenic epithelium associated with tooth development and account for approximately 0.19–2% of feline oral masses or oral tissue specimens [6,7,8,9].

Non-odontogenic cysts are distinguished from odontogenic cysts by their developmental origin, which is unrelated to tooth-forming tissues. Reports of such lesions in cats are extremely limited. Only a few cases have been described in young cats, including a nasopharyngeal dermoid cyst [10] and a nasopharyngeal cyst containing secretory proteinaceous material in a young cat [11].

Dermoid cysts are characterized by keratinous, sebum-like content or the presence of skin derivatives [12]. In humans, dermoid cysts arising on the floor of the mouth and other intraoral sites are histologically classified according to the Meyer classification [13], which distinguishes three types based on cyst tissue components. Epidermoid cysts are lined with stratified squamous epithelium and contain keratinaceous material but lack skin adnexal structures. True dermoid cysts contain skin appendages, such as sweat glands, sebaceous glands, and hair follicles. Teratoid cysts arise from totipotent cells and contain tissues derived from all three embryonic germ layers, including muscles, bones, cartilage, and various epithelial tissues [5,14]. In veterinary medicine, dermoid cysts arising at various anatomical sites have been described and classified according to the nature of the epithelial lining and cyst contents [15,16]. However, reports of these cysts arising within the oral cavity are extremely limited [10,17,18,19], and no widely accepted classification system specific to oral epidermoid, true dermoid, and teratoid cysts has been established in veterinary medicine.

Surgical excision is generally considered the treatment of choice for epidermoid cysts [5,10,11,13,17,18,19]. This report describes an epidermoid cyst arising from the soft palate of a geriatric cat, including its diagnostic imaging, histopathological findings, and surgical management.

## 2. Case Description

A 17-year-old, 2.82 kg, neutered female domestic shorthair cat presented with mild hypersalivation and dysphagia. The cat had a history of multiple dental extractions. Seventeen months before presentation, teeth 308, 309, 408, and 409 were extracted because of gingivitis associated with dental calculus. At that time, teeth 304, 308, 309, 404, 408, 409, 103, and 203 were present. No oral lesions suggestive of generalized stomatitis were identified other than gingivitis surrounding the teeth affected by dental calculus. After these extractions, teeth 304, 404, 103, and 203 remained. The cat had a 2-year history of hyperthyroidism managed with carbimazole and a 7-year history of chronic constipation managed with a high-fiber prescription diet and occasional enemas. Seizure episodes had developed 3 months before presentation and were being managed with levetiracetam. Comprehensive diagnostic evaluation was performed to assess the overall health status of the cat. Systolic blood pressure was 160 mmHg, and thoracic auscultation revealed no abnormalities. Hematologic, serum biochemical, and blood gas analyses revealed mild elevations in NT-proBNP (155.7 pmol/L, RI 0–100 pmol/L) and fPLI (3.8 μg/L, RI 0–3.5 μg/L), as well as hypernatremia (160 mmol/L, RI 145–151 mmol/L). Total thyroxine (T4) concentration was within the reference range. No other clinically relevant abnormalities were identified in blood tests.

Thoracic radiographs were unremarkable, whereas abdominal radiographs revealed fecal distension of the descending colon and rectum. Abdominal ultrasonography identified splenic and hepatic lesions, and cytology of the splenic lesion was suggestive of mast cell proliferation, prompting concurrent splenectomy and liver biopsy.

**Figure 1:**
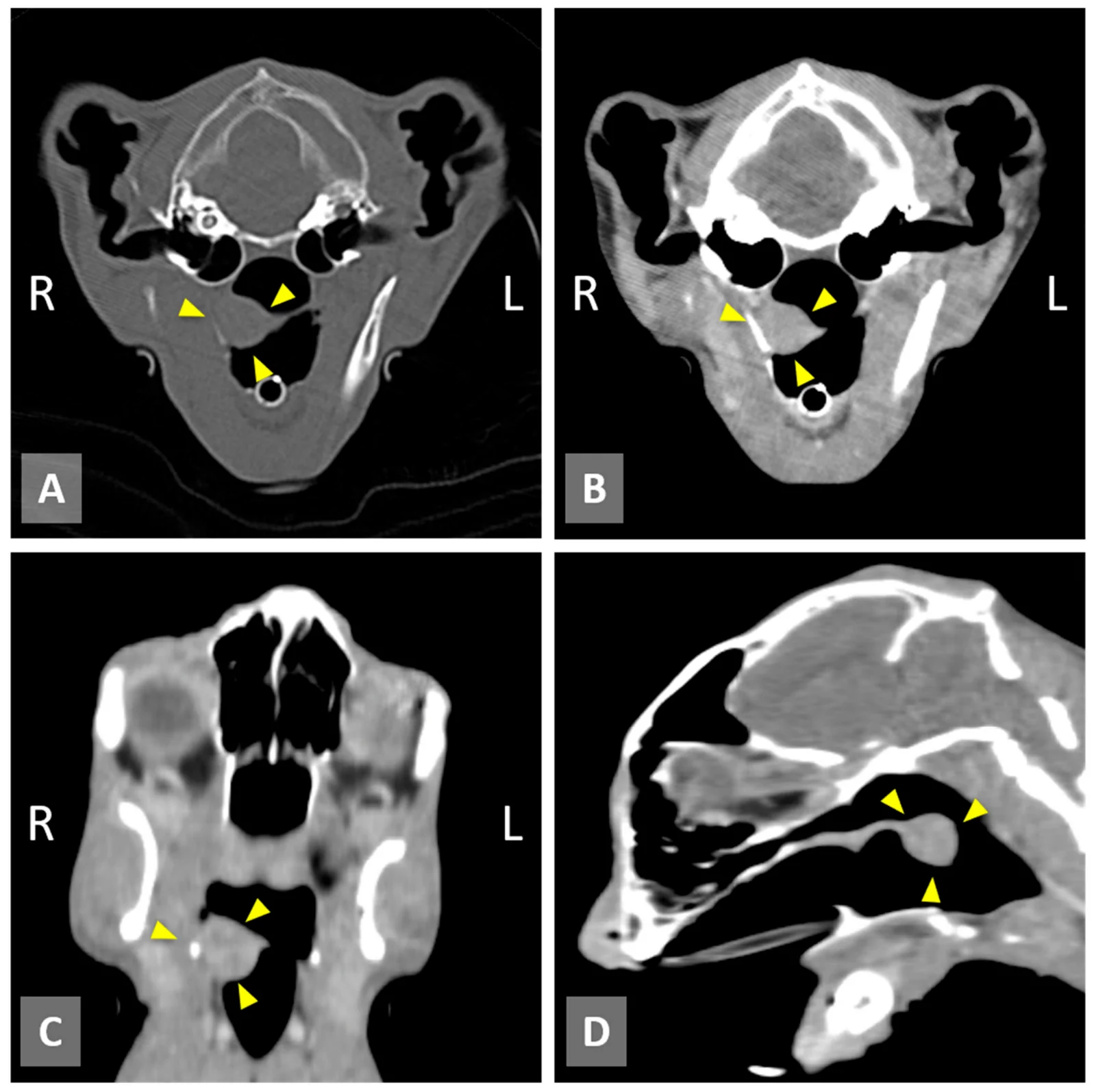
Computed tomographic images of the soft palate mass. (**A**) Pre-contrast axial (bone window), (**B**) post-contrast axial, (**C**) dorsal, and (**D**) sagittal images (soft tissue window) show a well-circumscribed polypoid mass (arrowhead) arising from the right distal soft palate and protruding predominantly into the oropharynx, with minimal nasopharyngeal obstruction. The mass showed minimal contrast enhancement (96.09 ± 3.46 HU pre-contrast; 102.11 ± 4.27 HU post-contrast; mean ± SD).

Head, thoracic, and abdominal computed tomography (CT) were performed for tumor staging and to evaluate the potential intracranial causes of the seizures. For the CT examination, the cat was premedicated with butorphanol (0.2 mg/kg, IV). Anesthesia was induced with propofol (6 mg/kg, IV, to effect) and maintained with isoflurane in oxygen. There was no evidence of metastases or intracranial lesions. However, CT incidentally revealed a well-circumscribed, polypoid mass (8.5 × 9.9 × 10.0 mm) arising from the right distal soft palate and extending into the oropharyngeal lumen, causing adjacent nasopharyngeal narrowing (Figure 1). No signs of bony invasion or osteolysis were observed in the adjacent osseous structures, making an odontogenic origin less likely. Following intravenous administration of iohexol (2.5 mL/kg), the mass exhibited minimal contrast enhancement, with mean attenuation values of 96.09 ± 3.46 HU on pre-contrast images and 102.11 ± 4.27 HU on post-contrast images. The relatively high pre-contrast attenuation and minimal contrast enhancement made a simple fluid-filled cyst or an enhancing solid neoplasm less likely. Nevertheless, inflammatory processes, including abscess and granuloma, as well as other neoplasia, could not be excluded based on the CT findings alone.

**Figure 2:**
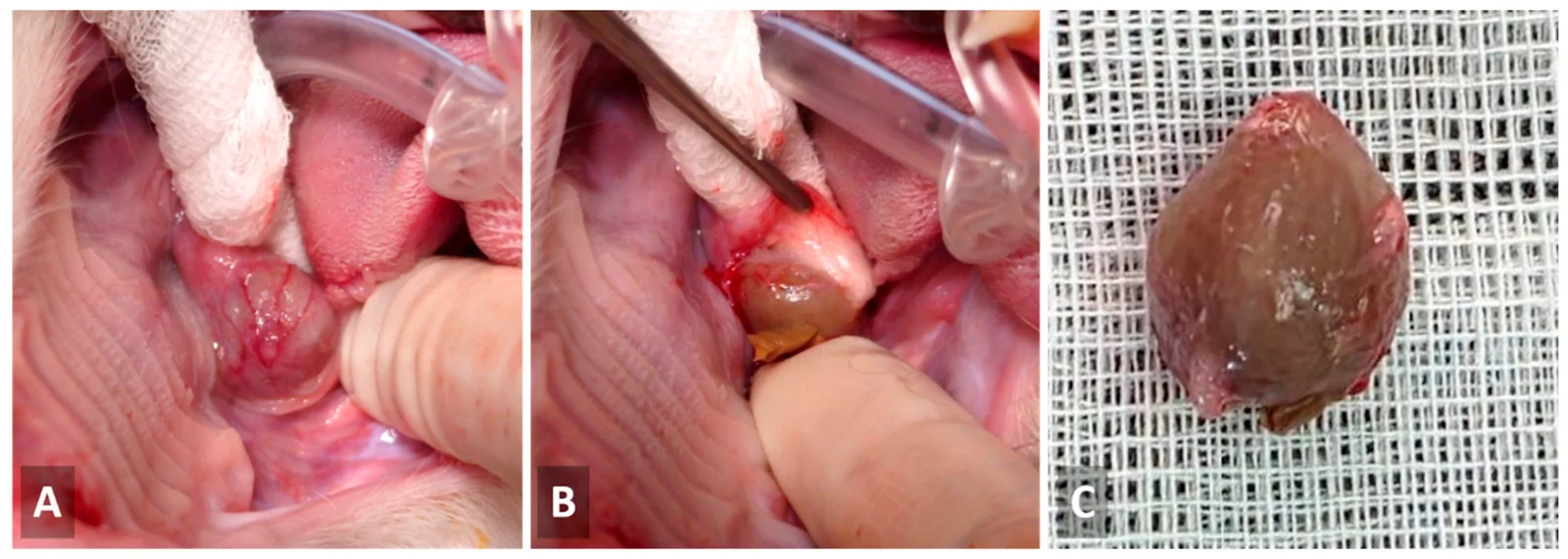
Soft palate mass in the cat. (**A**) Smooth and dome-shaped mass covered by mucosa. (**B**) Mucosal incision and blunt dissection of the mass. (**C**) Excised mass contains yellow-brown, creamy material.

Surgical excision of the soft palate mass was performed concurrently with splenectomy and liver biopsy under general anesthesia. Prior to anesthesia, the cat was clinically stable, and intravenous fluid therapy was administered to prevent dehydration and correct electrolyte abnormalities. The cat was premedicated with butorphanol (0.2 mg/kg, IV), while cefazolin (30 mg/kg, IV) and levetiracetam (20 mg/kg, IV) were administered perioperatively. Anesthesia was induced with propofol (6 mg/kg, IV) and maintained with inhaled isoflurane. Thorough examination of the oral cavity revealed no other oral or dental abnormalities. The mass was not readily visible on oral examination; however, digital palpation revealed a firm, mildly protruding lesion in the right oropharyngeal region. The lesion was grasped using atraumatic forceps and gently retracted rostrally, demonstrating good mobility and allowing clear visualization. Gauze packing was placed caudal to the mass to displace it rostrally and improve surgical exposure. The mass was covered with mucosa and had a smooth, dome-shaped surface (Figure 2A). A mucosal incision was made using a No. 15 scalpel blade, followed by blunt dissection of the mass from the surrounding tissue using a mosquito hemostat, iris scissors, and cotton-tipped applicators (Figure 2B). The mucosa was closely adhered to the underlying mass, which contained a yellow-brown creamy material (Figure 2C). Following grossly complete removal of the mass, the surgical site showed minimal hemorrhage and was left unsutured for second-intention healing.

Given the gross appearance of the mass contents, an infectious or inflammatory lesion was considered a differential diagnosis. To investigate a potential infectious etiology, the Feline Fever of Unknown Origin RealPCR Panel (IDEXX Laboratories, Inc., Westbrook, ME, USA) and the Feline Respiratory Pathogens Plus Panel and bacterial culture (GreenVet Laboratory, Yongin, Republic of Korea) were performed. The two panels included infectious agents potentially associated with the oral cavity (Supplementary Table S1). All PCR assays and the bacterial culture yielded negative results. Therefore, an infectious etiology of the lesion was considered unlikely.

**Figure 3:**
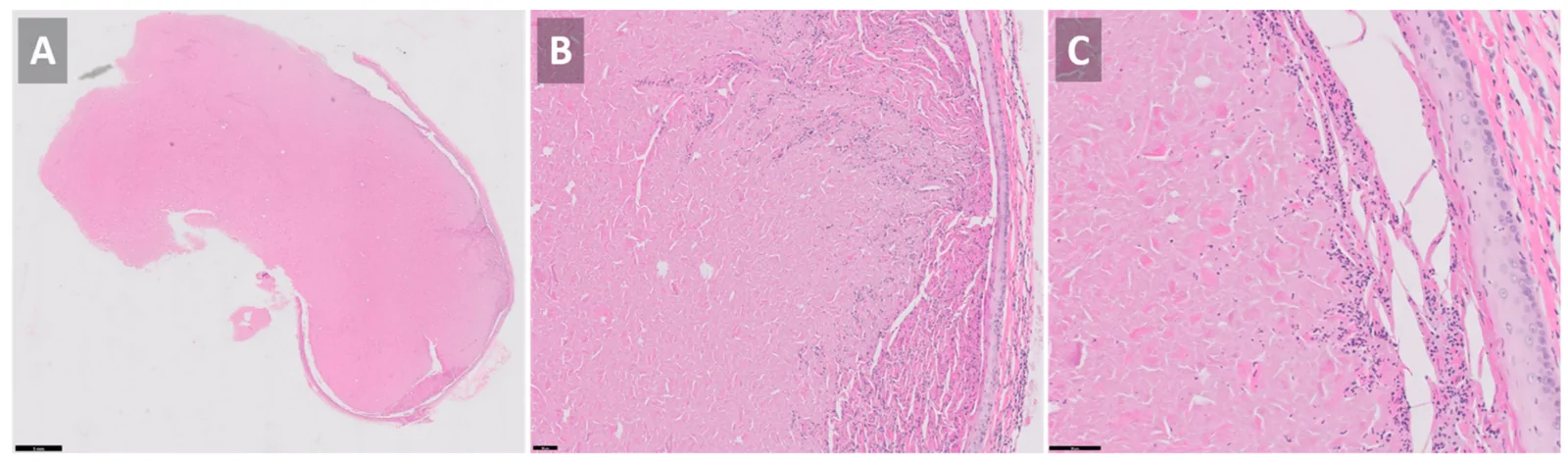
Histopathological findings of the soft palate mass in the cat. The cystic structure is lined by stratified squamous epithelium and contains luminal keratinaceous debris, granular eosinophilic material, and degenerate nuclear debris. (**A**) ×1, scale bar = 1 mm; (**B**) ×20, scale bar = 50 μm; (**C**) ×40, scale bar = 50 μm. Hematoxylin and eosin stain.

Histopathological examination (IDEXX Laboratories, Inc., Westbrook, ME, USA) confirmed multifocal atypical mast cell proliferation in the spleen and hepatocellular nodular hyperplasia in the liver. The soft palate mass was identified as a cyst lined with keratinizing stratified squamous epithelium of relatively uniform thickness, ranging from 2 to 5 cell layers (Figure 3). Keratinization was exclusively orthokeratotic and keratohyalin granules were not prominent. The lining epithelium was extensively bordered by compact, mature, reactive, and dense fibrovascular connective tissue. The lumen of the cyst was filled with granular eosinophilic material, keratinaceous debris with entrapped acicular cholesterol clefts, and mixed nuclear debris. Lesional tissue extended to the edges of the submitted specimen; histologically complete excision could therefore not be confirmed. These findings were consistent with those of a benign epidermoid cyst.

Postoperatively, the cat received cefazolin (30 mg/kg, q12h, IV), famotidine (2 mg/kg, q12h, IV), and butorphanol (0.2 mg/kg, q6h, IV) for three days. Because the soft-palate surgical site was left to heal by second intention and was exposed to the oral environment, short-term postoperative cefazolin administration was selected based on the patient’s individual risk factors, including advanced age, concurrent systemic disease, and concurrent abdominal surgery. This was a patient-specific clinical decision rather than routine postoperative antimicrobial prophylaxis for oral surgery. Butorphanol was administered for postoperative analgesia for three days. Additional analgesic options were limited by the patient’s advanced age, longstanding constipation, seizure disorder, and pre-existing renal abnormalities. Fentanyl was considered less desirable because of the patient’s gastrointestinal history, whereas NSAIDs were avoided because of the patient’s age and renal abnormalities. No additional analgesic was administered thereafter. Hypersalivation and dysphagia were no longer observed after surgery and voluntary food intake resumed; however, this improvement was documented over a period of only approximately 24 h, as marked gastric distension consistent with gastrointestinal hypomotility and recurrent seizures developed on the following day. Despite improvement in general condition and appetite, gastrointestinal dysmotility persisted and was associated with metabolic alkalosis, hypernatremia, and azotemia. Supportive treatment included ondansetron (0.1 mg/kg, q8h, IV), maropitant (1 mg/kg, q24h, IV), a continuous rate infusion (CRI) of metoclopramide (0.08 mg/kg/h, IV), parenteral nutrition, and fluid and electrolyte correction. The cat improved and was discharged on postoperative day 7. Three days after discharge, the cat was rehospitalized for recurrent vomiting, gastrointestinal hypomotility, and fecal retention, with transient improvement following enemas and metoclopramide CRI. Severe gastrointestinal distension recurred on postoperative day 23, necessitating nasoesophageal tube feeding. During the final hospitalization, a central venous catheter was placed to facilitate prolonged intensive supportive treatment, including parenteral nutritional support and intravenous fluid and electrolyte therapy. Two days later, profound gastrointestinal hypomotility and recurrent seizures developed, followed by severe vomiting, muscle tremors, metabolic alkalosis, and marked hypernatremia. Despite supportive treatment, the cat died on postoperative day 27.

## 3. Discussion

Epidermoid cysts have been reported in cats at various anatomical sites, including the intestine [20], lung [21], and intracranial cavity [22,23,24]. In the oral cavity, only one soft palate cyst with gross and histopathological features similar to those of this case has been reported in a young cat [11]. In humans, oral epidermoid cysts are developmental or acquired in origin [5,13]. Developmental cysts result from the entrapment of ectodermal tissue during embryonic fusion, whereas acquired cysts may arise from traumatic or iatrogenic implantation of the epithelium into deeper tissues [5,13]. Similar mechanisms have been proposed in veterinary medicine. A previously reported soft palate cyst in a young cat was suspected to be congenital, arising during the fusion of the palatine plates as they grew medially from the maxillary processes to form the palate [11]. Conversely, acquired lesions may develop secondary to inflammation or trauma in an older dog [17].

Previously, the nasopharyngeal epidermoid cyst extended into the tympanic bulla [17], resembling reported cases of aural cholesteatoma with oropharyngeal involvement in dogs and cats [25,26,27]. Cholesteatomas share similar histopathological features with epidermoid cysts in some respects. Compared with primary oral epidermoid cysts, cholesteatomas involving the oropharynx originate within the tympanic bulla, with subsequent extension into the oropharynx or intracranial cavity as the disease progresses [28]. Thus, the involvement of the middle ear is an important feature that distinguishes these lesions from primary oral epidermoid cysts. In this case, the cyst was predominantly confined to the soft palate with no evidence of abnormalities or continuity with the middle ear structures on CT. The present case provides additional clinical, diagnostic imaging, surgical, and histopathological information on a soft-palate epidermoid cyst in a geriatric cat.

Several cystic or cyst-like lesions were considered in the differential diagnosis. A salivary mucocele was considered less likely because the present lesion was a discrete, epithelial-lined cyst containing keratinaceous material, rather than a mucus-filled cavity associated with salivary tissue or extravasated saliva. An odontogenic keratocystic lesion was also considered because of the history of dental extraction. However, the absence of adjacent osseous involvement on CT and the lack of characteristic luminal parakeratinization and basal cell palisading on histopathology made this diagnosis less likely [9]. Infundibular keratinizing cystic lesions were also considered; however, the absence of adnexal structures and the anatomic location within the soft palate were not supportive of a follicular or cutaneous adnexal origin. Cystic or keratinizing squamous cell carcinoma was considered as a neoplastic differential diagnosis, but the absence of cellular atypia, invasive epithelial growth, and malignant keratinization on histopathological examination did not support this diagnosis [1,2,3].

Although an acquired origin was considered as a possible explanation, the origin of the cyst could not be determined. A developmental cyst that remained clinically silent until old age cannot be excluded. In humans, traumatic implantation of epithelial cells into deeper tissues has been proposed as a mechanism for the development of acquired epidermoid cysts [29]. In an in vivo rat model, subcutaneous implantation of autogenous skin resulted in the proliferation of the implanted epithelium and formation of epithelium-lined cystic cavities containing keratin, hair, and other debris, resembling epidermoid or dermoid cysts [30].

Although there was no known history of direct trauma in this case, an acquired origin associated with previous mucosal injury was considered as one possible explanation. The cat had undergone caudal mouth extraction under general anesthesia with endotracheal intubation 17 months before the cyst was identified. Although the cat had a history of dental extractions and continued consumption of dry food, there was no documented evidence linking these factors to mucosal injury or cyst formation [29]. Therefore, none of these exposures can be considered evidence of a causal relationship, and the proposed acquired mechanism remains speculative.

Previously reported nasopharyngeal cysts in cats were associated with respiratory signs and marked nasopharyngeal obstruction [10,11]. Nasopharyngeal polyps were also considered because of the anatomical location. Feline nasopharyngeal polyps are benign inflammatory fibrovascular proliferations, typically arising from the middle ear or auditory tube and covered by epithelium. Histologically, they consist of fibrovascular connective tissue with inflammatory cells rather than a discrete keratin-filled cyst lined by stratified squamous epithelium [31]. In the present case, the lesion was a well-circumscribed, keratin-filled cyst lined by stratified squamous epithelium and arising from the distal soft palate, while CT showed no evidence of middle-ear or tympanic bulla involvement, making a nasopharyngeal polyp unlikely. The cat also showed no apparent respiratory signs, which may have been related to the small size of the cyst and its predominant confinement to the oropharynx, resulting in minimal nasopharyngeal obstruction. Furthermore, the lesion was detected incidentally on cranial CT, potentially before it had sufficiently enlarged to cause clinically relevant nasopharyngeal obstruction.

Mild hypersalivation and dysphagia were present before surgery and were considered possibly related to the soft palate lesion. Following excision, neither sign was observed during periods of clinical stability. Other potential explanations were also considered. No other oral or dental abnormalities that could readily account for them were identified on thorough examination of the oral cavity under general anesthesia or on CT. Pancreatic abnormalities and increased fPLI had been identified as chronic findings before surgery, without substantial changes in laboratory or imaging findings postoperatively, making acute pancreatitis-associated nausea a less likely explanation. Azotemia was not present preoperatively and therefore was unlikely to account for the initial signs. Hyperthyroidism and potential adverse effects of carbimazole and levetiracetam were also considered less likely because the same medications were continued after surgery without recurrence of hypersalivation or dysphagia during clinically stable periods. In contrast, hypernatremia was documented both preoperatively and during subsequent episodes of gastrointestinal dysfunction accompanied by vomiting and therefore could not be excluded as a contributing factor. Although interpretation of the postoperative improvement was confounded by concurrent splenectomy and perioperative analgesic and gastroprotective treatment, the temporal association between excision and resolution of hypersalivation and dysphagia suggests that the soft palate lesion may have contributed to these signs. Despite its small size and relatively mild degree of obstruction, the lesion may have caused local discomfort or interfered with swallowing.

Surgical excision is recommended for epidermoid cysts regardless of their anatomical location [10,11,17,20,21,22,23,24,29,32,33,34,35]. Recurrence following surgical excision is uncommon in veterinary patients; however, recurrence has been reported in a dog with an extradural spinal epidermoid cyst, in which the cyst regrew with recurrence of clinical signs 28 months after subtotal excision [34]. Similarly, recurrence is uncommon in humans and associated with incomplete excision [36,37]. In the present case, histologically complete excision could not be confirmed; however, no residual lesion was evident macroscopically after excision, and therefore no further surgical intervention was performed. The short postoperative follow-up precluded assessment of subsequent recurrence. A recent long-term follow-up study of cerebellopontine angle epidermoid cysts found no association between the extent of resection and recurrence [38]. Therefore, the authors advocated prioritizing surgical safety over aggressive attempts at complete removal if complete excision carries a substantial risk of postoperative complications [38].

Interestingly, malignant transformation of epidermoid cysts into carcinoma has been reported in humans, although it is exceedingly rare [39,40,41,42,43,44,45,46,47]. This risk is higher in surgically untreated or incompletely excised lesions. Because recurrence and malignant transformation in humans typically take several years to develop, these outcomes may be underrecognized in veterinary medicine owing to the limited availability of long-term follow-up.

In this case, the initial seizure episodes had developed approximately three months before presentation, when the cat was clinically stable and no major electrolyte abnormalities had been identified. According to the owner, the episodes were frequently triggered by loud sounds, and audiogenic reflex seizures were considered a possible seizure phenotype. During the postoperative course, however, clinically significant electrolyte disturbances, including hypernatremia, recurred in association with gastrointestinal hypomotility and vomiting. Given the cat’s longstanding constipation and fecal distension at presentation, chronic dehydration may also have contributed to the pre-existing hypernatremia. Although these abnormalities were repeatedly corrected, increasingly frequent seizures occurred shortly before death and may have been exacerbated by the electrolyte disturbances. Thus, pre-existing hypernatremia and possible chronic dehydration may have contributed to the preoperative clinical signs and may also have exacerbated the terminal clinical course; however, their specific contribution cannot be determined from the available clinical data. The splenic mast cell proliferation had been surgically removed during the initial procedure, with no clinical evidence of recurrence thereafter. Nevertheless, occult visceral mast cell neoplasia could not be excluded as a potential contributor to the deterioration. Based on the clinical course, the deterioration appeared to be more closely associated with gastrointestinal dysfunction following the concurrent abdominal surgery than with the soft-palate procedure itself. However, because the owner declined postmortem examination after several hospitalizations, this attribution remains speculative, and the cause of death could not be established. Other complications, including occult infection, also cannot be completely excluded.

## 4. Conclusions

We describe a soft-palate epidermoid cyst in a geriatric cat, with detailed clinical, diagnostic imaging, surgical, and histopathological findings. The lesion was predominantly confined to the oropharyngeal aspect of the soft palate and showed no evidence of middle-ear or bony involvement. Hypersalivation and dysphagia were no longer observed during the short postoperative period; however, this temporal association does not establish a causal relationship, and long-term outcome could not be assessed because of subsequent fatal gastrointestinal and systemic complications. The origin of the cyst remains uncertain, and both developmental and acquired mechanisms should be considered.

## Data Availability

The original contributions presented in this case report are included in the article. Further inquiries can be directed to the corresponding author.

## Supplementary Materials

The following supporting information can be downloaded at: https://www.mdpi.com/article/10.3390/vetsci13101064/s1, Table S1. Infectious agents included in the commercial diagnostic PCR panels used in this case.

## Abbreviations

The following abbreviations are used in this manuscript:

CT	Computed Tomography
CRI	Continuous Rate Infusion
fPLI	Feline Pancreatic Lipase Immunoreactivity
NT-proBNP	N-terminal pro-B-type Natriuretic Peptide
PCR	Polymerase Chain Reaction
RI	Reference Interval
T4	Thyroxine
