# Supplementary material for: Epidermoid Cyst of the Soft Palate in a Geriatric Cat: A Case Report
Source: Vet Sci. 2026 Oct 9;13(10):1064. doi: 10.3390/vetsci13101064 (PMC13709755; doi:10.3390/vetsci13101064)
Supplement: Supplementary file 1 [file vetsci-13-01064-s001.zip › vetsci-4553237-supplementary.pdf]

Supplementary Table S1. Infectious agents included in the commercial diagnostic PCR panels used in this case.

Panel	Feline Fever of Unknown Origin RealPCR Panel*	Feline Respiratory Pathogens Plus Panel†
Target pathogen	feline calicivirus, feline coronavirus, feline leukemia virus, feline immunodeficiency virus, feline panleukopenia virus, *Bartonella spp., Anaplasma spp., Ehrlichia spp., Mycoplasma haemofelis,* Candidatus *Mycoplasma haemominutum, Salmonella spp.,* Candidatus *Mycoplasma turicensis, Cryptococcus spp., Cytauxzoon felis, Toxoplasma gondii*	feline calicivirus, influenza A virus, reovirus, feline herpesvirus, *Bartonella spp., Pasteurella multocida, Moraxella catarrhalis, Chlamydophila felis, Bordetella bronchiseptica, Pneumocystis carinii* f. sp. *felis, Mycoplasma felis, Cryptococcus spp., Aspergillus spp., Histoplasma spp., Blastomyces spp.*

* IDEXX Laboratories, Inc., Westbrook, ME, USA

† GreenVet Laboratory, Yongin, Republic of Korea
